# The transcriptional response of microbial communities in thawing Alaskan permafrost soils

**DOI:** 10.3389/fmicb.2015.00197

**Published:** 2015-03-16

**Authors:** Marco J. L. Coolen, William D. Orsi

**Affiliations:** ^1^Marine Chemistry and Geochemistry Department, Woods Hole Oceanographic InstitutionWoods Hole, MA, USA; ^2^Western Australia Organic and Isotope Geochemistry Centre, Department of Chemistry, Curtin UniversityPerth, WA, Australia

**Keywords:** permafrost, metatranscriptomics, hydrolysis of biopolymers, acetoclastic methanogenesis, biofilm formation, DNA repair, cellular defense mechanisms

## Abstract

Thawing of permafrost soils is expected to stimulate microbial decomposition and respiration of sequestered carbon. This could, in turn, increase atmospheric concentrations of greenhouse gasses, such as carbon dioxide and methane, and create a positive feedback to climate warming. Recent metagenomic studies suggest that permafrost has a large metabolic potential for carbon processing, including pathways for fermentation and methanogenesis. Here, we performed a pilot study using ultrahigh throughput Illumina HiSeq sequencing of reverse transcribed messenger RNA to obtain a detailed overview of active metabolic pathways and responsible organisms in up to 70 cm deep permafrost soils at a moist acidic tundra location in Arctic Alaska. The transcriptional response of the permafrost microbial community was compared before and after 11 days of thaw. In general, the transcriptional profile under frozen conditions suggests a dominance of stress responses, survival strategies, and maintenance processes, whereas upon thaw a rapid enzymatic response to decomposing soil organic matter (SOM) was observed. Bacteroidetes, Firmicutes, ascomycete fungi, and methanogens were responsible for largest transcriptional response upon thaw. Transcripts indicative of heterotrophic methanogenic pathways utilizing acetate, methanol, and methylamine were found predominantly in the permafrost table after thaw. Furthermore, transcripts involved in acetogenesis were expressed exclusively after thaw suggesting that acetogenic bacteria are a potential source of acetate for acetoclastic methanogenesis in freshly thawed permafrost. Metatranscriptomics is shown here to be a useful approach for inferring the activity of permafrost microbes that has potential to improve our understanding of permafrost SOM bioavailability and biogeochemical mechanisms contributing to greenhouse gas emissions as a result of permafrost thaw.

## Introduction

The northern permafrost region contains ~1672 gigatonnes (Gt) of organic carbon, nearly twice the amount of atmospheric carbon. Roughly 1470 Gt or 88% of this carbon occurs in perennially frozen soils and deposits (Tarnocai et al., 2009). The majority of permafrost organic matter is derived from (partially decomposed) plant material and was buried by dust deposition, sedimentation in flood plains and peat development on time scales of decades to millennia (Zimov et al., 2006; Schuur et al., 2008). Permafrost is overlain by a soil horizon that varies in thickness from a few centimeters to several meters, which experiences seasonal freeze–thaw cycles (i.e., the active layer). Owing to atmospheric warming, the depth of the active layer has been increasing in many locations over the past few decades, resulting in a subsequent decline in the underlying permafrost (Kane et al., 1991; Anisimov et al., 1999; Jorgenson et al., 2001; Zhang et al., 2005; Pautler et al., 2010). This warming-induced thickening of the active layer is expected to enhance microbially mediated soil organic matter (SOM) decomposition, and release of carbon from the upper permafrost to the atmosphere (Dutta et al., 2006; Uhlirova et al., 2007; Schuur et al., 2008; Pautler et al., 2010).

Despite subfreezing temperatures and low water availability, permafrost soils up to 3 million years in age harbor a large diversity of bacteria, archaea, and fungi as revealed by sequencing of environmental ribosomal RNA (rRNA) genes (Vishnivetskaya et al., 2006; Liebner et al., 2008; Waldrop et al., 2010; Coolen et al., 2011; Penton et al., 2013; Bakermans et al., 2014; Frank-Fahle et al., 2014; Ganzert et al., 2014). A variety of microbes have retained viability in frozen permafrost over geological time periods and, upon thawing, could renew or accelerate their physiological activity. Many permafrost bacterial isolates are cold-adapted heterotrophs belonging to the phyla Firmicutes, Actinobacteria, Proteobacteria, and Bacteroidetes, while many of the archaeal isolates are methane (CH_4_)-producers (methanogens) growing on hydrogen (H_2_) and carbon dioxide (CO_2_) with some species also growing on formate, methanol, or methylamine (Jansson and Tas, 2014 and references therein).

Genomes of bacterial isolates have also revealed insights into strategies for survival in subzero conditions. For example, the genome of the Siberian permafrost isolate *Psychrobacter arcticus* 273-4 revealed three cold shock proteins, which operate as RNA chaperones that enhance translation processes by eliminating the formation of secondary structures in the messenger RNA (mRNA) (Ayala-Del-Rio et al., 2010). This adaptation is important for *P. arcticus*, and possibly other permafrost bacteria, because subzero temperatures make mRNA more stable and less efficient for translation. Furthermore, *P. arcticus* uses acetate, which can diffuse into the cell without an energetically costly transport system, as the basis for its biosynthesis and energy metabolism (Ayala-Del-Rio et al., 2010). However, cultured species isolated from permafrost are not necessarily representative of active microbial community *in situ*, and the ability of some members to form spores (e.g., Firmicutes) may bias phylogenetic representation in culture (Steven et al., 2008; Niederberger et al., 2009).

Recent pioneering metagenomic studies have helped to circumvent some cultivation-biased interpretations of *in situ* activity, and revealed cultivation-independent insights into which biochemical pathways are present and potentially expressed by permafrost microbiota. Overall, these studies suggest that permafrost microbial communities have a large metabolic potential for carbon processing, including pathways for fermentation, methanogenesis, and nitrogen cycling (Yergeau et al., 2010; Mackelprang et al., 2011; Lipson et al., 2013). However, metagenomic surveys do not provide information on the relative importance and the exact timing of biochemical processes, as cells need to replicate their genomes first to monitor an increase in metabolic potential. In addition, in pristine frozen soil samples it is difficult to distinguish between genes involved in ongoing vs. past microbial processes using DNA-based metagenomic datasets because bacterial, fungal, and plant DNA can be preserved for thousands of years in permafrost soils (Willerslev et al., 2003, 2004; Bellemain et al., 2013). However, metatransciptomic analysis of extremely short-lived mRNA would provide information on microbial activities that occurred in the permafrost soils at the time of sampling.

Here, we performed a pilot study using ultrahigh throughput Illumina HiSeq sequencing of reverse transcribed mRNA (e.g., Orsi et al., 2013; Huang et al., 2014) to obtain a detailed overview of active metabolic pathways and responsible organisms in permafrost soils under pristine frozen conditions and transcriptional responses after 11 days of thaw. The permafrost soil horizons analyzed (up to 70-cm-deep) are expected to thaw in the Alaskan Arctic within decades as a result of continuing Arctic warming (Osterkamp, 2007). For our analysis, we were particularly interested in the expression of biochemical pathways that are hypothesized to play an important role in mediating the release of greenhouse gasses from thawing permafrost (e.g., CO_2_ and CH_4_). We sought to provide the first transcriptional data supporting the hypothesis that microbial degradation of SOM biopolymers leads to increased CO_2_ production and methanogenesis, and to elucidate the biochemical mechanisms underlying these processes. Parallel geochemical analysis of soil age, carbon and nitrogen content, and lipid biomarkers provided additional information on the sources and composition of permafrost SOM.

## Methods

### Site description and sample collection

Using a SIPRE style auger system (Jon's Machine Shop, Fairbanks, AK), a 130-cm-long (8 cm diameter) core was recovered on July 27, 2008 from moist acidic tundra (MAT) near the Kuparuk River at the Toolik Long Term Ecological Research (LTER) Field Station, Alaska (68°38′41.455″N:149°24′09.682″W) (Coolen et al., 2011). The pH of the soils was not determined, but a pH of 3–4 has been reported from comparable North Alaskan MAT soils (Hobbie and Gough, 2004). According to the Alaska Tundra Vegetation Map (Walker and Maier, 2008), the coring location was located in bioclimate subzone E where mean July temperatures are between 9 and 12°C and the tundra vegetation covering the coring site is defined as Class 4.1 Tussock-sedge, dwarf shrub, moss communities on mesic, acidic loess. This is the most common type of vegetation, covering 100,000 km^2^ or 25% of the land surface in Arctic Alaska. The thickness of the active layer was 26 cm at the time of coring. Core sections were described in the field and immediately wrapped in sterile, baked (500°C, 8 h) aluminum foil and kept frozen inside coolers with deeply frozen blue ice packs. At the Toolik Field Station, the melted active layer was kept inside the cooler and the frozen core sections were kept in a freezer at −5°C for up to 2 h until subsampling was completed: within 2 h after coring an uncontaminated subsampling surface area was created by removing the outer 1 cm of frozen soil with a sterile knife. Cores were split laterally at the desired depths using a sterilized hammer and a chisel, and subsamples were obtained only from the central part of each interval with sterilized scalpel and tweezers. Thus, the distance between the core liner and subsampled sediment was at least 2 cm to avoid cross contamination.

Soil samples were collected immediately after coring on July 27, 2008 from two active layer horizons (12 ± 2 and 24 ± 2 cm), and five depth intervals from the underlying permafrost (33.5 ± 2, 49 ± 2, 68 ± 2, 109 ± 2, and 126 ± 2 cm). For lipid analysis, subsamples (~2 g) were stored frozen at −20°C at the Woods Hole Oceanographic Institution (WHOI) until subsequent lipid extraction. For the subsequent extraction of RNA, ~2 g aliquots of pristine soils were transferred to 5 ml cryovials and immediately flash frozen in liquid nitrogen. In addition, 20 ml sterile serum flasks were completely filled with soil leaving no headspace, capped airtight, and incubated in the dark at 4°C. As samples were capped airtight with no headspace, they remained anoxic for the duration of the incubation. Subsamples for RNA extraction were also obtained on the last day of fieldwork (i.e., after 11 days of thaw/incubation). All samples for RNA work were stored in liquid nitrogen during airfreight transport to WHOI and RNA was extracted immediately upon arrival as detailed below.

**Figure 1:**
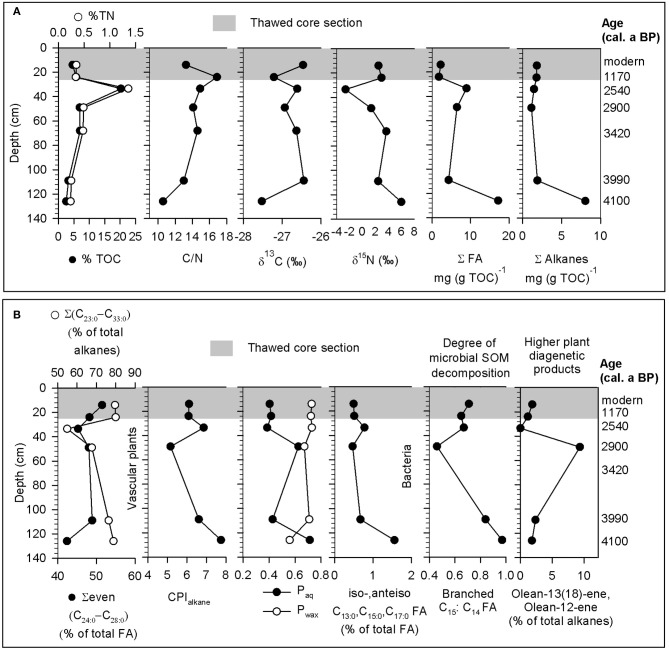
**Geochemistry of SOM in the 2 active layer (14 ± 2 and 24 ± 2 cm) and 5 permafrost soil horizons (33.5 ± 2 to 126 ± 2 cm) of core TL08S1C3. (A)** Bulk geochemical and stable isotope data and total concentrations of FAs and alkanes. **(B)** Percent contributions and ratios from lipid sub-classes representing different biological sources.

Radiocarbon dates and bulk geochemical analysis (%TOC, %TN, δ^13^C, δ^15^N) from the exact same intervals were provided previously (Coolen et al., 2011), and compared with the lipid biomarker results from the present study (Figure 1).

### Soil organic matter composition

Lipid biomarker compounds were extracted from soils collected from the 7 depth horizons with a methylene chloride:methanol mixture (9:1, v:v) using a microwave-accelerated reaction system (MARS6) (CEM, Matthews, NC) in which samples were heated at 100°C for 15 min with continuous stirring (Osburn et al., 2011). Following extraction, samples were filtered, dried under N_2_ gas, and saponified with aqueous 0.5 M sodium hydroxide at 70°C for 4 h. Cooled samples were acidified with 3N hydrochloric acid (pH < 3) before being extracted 3 times with methyl tert-butyl ether. Subsequently, elemental sulfur was removed by filtering extracts through acid-washed copper. Samples were resuspended in hexane prior to separation of compound classes by solid phase extraction. Discovery DSC-NH2 stationary phase (Sigma-Aldrich, St. Louis, MO) (1 g) was packed into glass columns and samples were separated into five fractions: F1 5 ml hexane; F2 8 ml of 4:1 hexane/methylene chloride; F3 10 ml of 9:1 methylene chloride/acetone; F4 15 ml of 2% formic acid in methylene chloride (Sessions, 2006). Fatty acids (F4) were methylated with acidic methanol (95:5 methanol/HCl) and heated overnight at 70°C to form fatty acids methyl esters. Fractions containing hydrocarbons (F1) and fatty acids (F4) were concentrated and an internal standard was added prior to analysis by gas chromatography—mass spectrometry (GC-MS). One microliter of sample was injected into an Agilent 7890 GC (Agilent Technologies, Santa Clara, CA) with an effluent split ~70:30 between a 5975C mass spectrometer and a flame ionizing detector. Peaks were separated on a DB-5 ms column (60 m long, 0.25 mm ID, 0.25 μm film thickness). Data from hydrocarbon (F1) and fatty acid (F4) fractions are presented here for all samples except the 68 cm horizon, due to sample loss. From *n*-alkane concentrations (hydrocarbon fraction) we calculated the carbon preference index (CPI), P_aq_ index, and P_wax_ index. CPI_alkane_ reflects relative inputs of higher plants vs. algae and bacteria since the former have a strong odd/even predominance. Consequently, high CPI_alkane_ values (>5) reflect mainly contributions from higher plants (Cranwell et al., 1987).

$$\begin{array}{l} {\text{CPI}}_{\text{alkane}}=\;(\Sigma {\left({\text{C}}_{21}-{\text{C}}_{29}\right)}_{\text{odd}}+\Sigma {\left({\text{C}}_{23}-{\text{C}}_{31}\right)}_{\text{odd}})/\\ \;\;\;(2^{*}\Sigma {\left({\text{C}}_{22}-{\text{C}}_{30}\right)}_{\text{even}}). \end{array}$$

The P_aq_ index reflects relative contributions of alkanes from aquatic macrophytes vs. higher plants (Ficken et al., 2000). High P_aq_ values (>0.5) indicate inputs from submerged or floating macrophytes and may reflect wet conditions.

$${\text{P}}_{\text{aq}}=\;({\text{C}}_{23}+{\text{C}}_{25})/({\text{C}}_{23}+{\text{C}}_{25}+{\text{C}}_{29}+{\text{C}}_{31}).$$

A high P_wax_ index value (>0.7) reflects waxy inputs from higher plants and may indicate drier conditions (Zheng et al., 2007):

$${\text{P}}_{\text{wax}}=\;({\text{C}}_{27}+{\text{C}}_{29}+{\text{C}}_{31})/({\text{C}}_{23}+{\text{C}}_{25}+{\text{C}}_{27}+{\text{C}}_{29}+{\text{C}}_{31}).$$

### RNA extraction and purification

Immediately after arrival of the flash frozen soil samples at WHOI, RNA was extracted in triplicate from ~2 g of soil using the Power Soil™ Total RNA Isolation kit (MoBio Laboratories, Inc., Carlsbad, CA) following the instructions of the manufacturer. The homogenization step started while the soil was still frozen to minimize degradation of RNA during thawing of the flash frozen soil samples. Standard procedures to further minimize degradation of RNA during the extraction process involved the use of filter tips and certified RNAse free disposables and reagents. Surfaces were cleaned with RNase AWAY™ (Life Technologies, Grand Island, NY), and all extraction procedures were performed in a HEPA-filtered horizontal laminar flow hood (Labconco, Kansas City, MO) inside a HEPA-filtered and positive-pressured ancient DNA dedicated lab at WHOI to eliminate aerosol contamination by bacterial and fungal cells/spores. DNA was removed using the Turbo DNA-free® kit (Life Technologies), increasing the incubation time to 1 h to ensure rigorous DNA removal. Extraction blanks were performed (adding sterile water instead of sample) to ensure that aerosolized contaminants did not enter sample and reagent tubes during the extraction process. Short RNA fragments (mostly produced during the extraction protocol) and residual inhibitors (i.e., humics) were removed from the extracted RNA using the MEGA-Clear® RNA Purification Kit (Life Technologies). We followed the protocol all the way through the optional precipitation/concentration step, resuspending the RNA pellet in 10 μl of certified RNAse free sterile water (Life Technologies). Prior to cDNA amplification, the removal of contaminating DNA in RNA extracts was confirmed by the absence of visible amplification of SSU rRNA genes after 35 cycles of PCR using the RNA extracts as template, and the quality of the RNA was verified by agarose gel electrophoresis. All downstream analyses (cDNA amplification, sequencing, and bioinformatic analyses) described briefly in the following sections followed the protocols developed and reported recently (Orsi et al., 2013, 2015).

### Choice of samples for subsequent metatranscriptomic analysis

The permafrost soil horizons that were selected for cDNA amplification and Illumina sequencing (up to 70-cm-deep) are expected to thaw in the Alaskan Arctic as a result of continuing Arctic warming (Osterkamp, 2007). High quality RNA was extracted from the permafrost table (33.5 ± 2 cm) and from the horizon at 49 ± 2 cm before and after thaw. However, only after thaw the soil interval of 68 ± 2 cm yielded visible amounts of RNA on gel and subsequent cDNA amplification and Illumina sequencing was therefore not performed on the pristine frozen sample at this depth. The selected permafrost table was thought to contain relatively labile OM (Coolen et al., 2011) whereas the two deeper late-Holocene permafrost intervals (at 49 and 68 cm depth) potentially contained more recalcitrant SOM. Unfortunately, for budgetary reasons we were not able to sequence the metranscriptomes from the overlying active layers. However, parallel analysis of the microbial community composition (ribosomal RNA genes and transcripts) and microbial ectoenzyme activities involved in the hydrolysis of complex soil biopolymers, previously revealed that the most dramatic response in microbial activities and communities occurred in the permafrost intervals after 11 days of thaw while activities and microbial communities underwent little variation in the active layers over the course of incubation (Coolen et al., 2011). For the above reasons, subsequent Illumina sequencing of the reverse transcribed mRNA (this study) was only performed from depth horizons 33.5 ± 2 and 49 ± 2 cm (before and after 11 days of thaw) and 68 ± 2 cm (after 11 days of thaw).

### cDNA amplification and illumina sequencing

Since Illumina HiSeq sequencing was not yet available in 2008, the following steps were performed 5 years after frozen storage (−80°) of the purified RNA: Five nanograms of purified high quality RNA as determined fluorometrically with Quant-iT™ RiboGreen® RNA reagent (Life Technologies) was used as template for selective mRNA amplification using the Ovation RNA-Seq v2 System® (NuGEN technologies, San Carlos, CA). We followed the manufacturers instructions for cDNA amplification, and the resulting quantity of cDNA was checked fluorometrically using the Quant-iT™ PicoGreen® dsDNA Assay Kit (Life Technologies). Quality of the amplified cDNA was checked on a Bioanalyzer (Agilent Technologies, Santa Clara, CA) prior to Illumina® sequencing. cDNA of the triplicate extracts were pooled and Illumina® library preparation and paired-end sequencing was performed at the University of Delaware Sequencing and Genotyping Center (Delaware Biotechnology Institute, Newark DE). We acknowledge that because the original RNA extracts were stored ~5 years at −80°C prior to metatranscriptome construction, it is possible that some RNA could have been lost or partially degraded.

### Quality control and assembly

Quality control of the dataset was performed using FastQC (http://www.bioinformatics.babraham.ac.uk/projects/fastqc/), with a quality score cutoff of 28. Approximately 375 million paired-end reads that passed quality control were imported into CLC Genomics Workbench 6.0® (CLC Bio Inc., Cambridge, MA) and assembled using the paired-end Illumina assembler. Contigs were assembled over a range of kmer sizes (20, 50, 60, 64) with a minimum contig size cutoff of 300 nucleotides. The kmer size of 50 resulted in the highest number of contigs and thus these contigs were chosen for use in downstream bioinformatics analyses. To reduce the formation of chimeric assemblies, we used a 2 × 105 paired-end sequencing approach on the Illumina HiSeq platform and performed assemblies without scaffolding. Reads were mapped onto the contigs using the read mapping option in CLC Genomics Workbench to retain information on relative abundance of contigs.

### Functional annotation of contigs

Contigs were submitted to CAMERA (Community Cyber infrastructure for Advanced Microbial Ecology Research and Analysis, http://camera.calit2.net/) and assigned to clusters of orthologous gene (COG) families using the Rapid Analysis of Multiple Metagenomes with a Clustering and Annotation Pipeline (RAMMCAP) (Li, 2009) using the 6 reading frame translation option for open reading frame (ORF) prediction and BLASTn for rRNA identifications. The cutoff criterion *E*-value of 10^−5^ was used for RPS BLAST searches the COG database. For identification of bacterial and archaeal ORFs, the RAMMCAP analyses were performed using the bacterial and archaeal genetic code (-t 11 in advanced options). For identification of fungal ORFs, additional RAMMCAP analyses were performed using the standard genetic code for eukaryotes and the alternative yeast genetic code (−t 1 and −t 12 in advanced options). cDNA contigs were also submitted to MG-RAST (Meyer et al., 2008), and ORFs were detected and annotated according to their standard bioinformatics pipeline. Data have been deposited in in MG-RAST under accession numbers 4517418.3, 4517417.3, 4517416.3, 4517415.3, 4517414.3.

### Taxonomic annotation of contigs

Contigs were assigned to high-level taxonomic groups (Class level and above) using a Naïve Baysien Classifier (NBC) that compares against a database containing all DNA sequences in NCBI that classified as either Bacteria, Archaea, Fungi, or viral (Rosen and Essinger, 2010). This approach was chosen because NBC has been found to outperform most other composition, similarity, and phylogeny based metagenomic classifiers in terms of sensitivity and precision (Bazinet and Cummings, 2012). Custom perl scripts were used to merge NBC taxonomic identifications of contigs, with COG and Pfam annotations from RPS-BLAST searches together with average coverage (read mapping) from assemblies.

### Mapping of metatranscriptomic data to *M. barkeri* methanogenesis pathways

The bioinformatics pipeline for metatranscriptomic genome recruitment followed that described recently (Orsi et al., 2015). In brief, contigs from frozen and thawed metatranscriptomes were searched for homology in the genome of *M. barkeri* via BLASTx (e^−5^, >60 amino acid identity) searches using the translated amino acid sequences of *M. barkeri* genes as a reference. Expressed genes with homology *M. barkeri* proteins were subjected to metabolic pathway mapping in KEGG (http://www.genome.jp/kegg/pathway.html). Expressed genes with homology to the *M. barkeri* genome involved in methanogenesis pathways were visualized using Adobe Illustrator (Adobe Systems Inc., San Jose, CA).

### Analysis of gene overexpression in frozen vs. thawed soil samples

Analyses of gene overexpression in frozen vs. thawed samples was performed using the R statistical package (http://www.r-project.org/), with the MG-RAST matR library (metagenomics.anl.gov). To maintain abundance information, assembled contig sequences from each sample were uploaded to MG RAST with the read mapping abundance added to the fasta headers as specified on the MG RAST website. Differences in overexpression between frozen and thawed permafrost samples were determined by a One-Way ANOVA test with a *P*-value cutoff of 0.025. The relatively low number of rRNA reads that were present in the dataset despite the selective amplification of mRNA using the Ovation RNA-Seq v2 System® were removed prior to comparison. Data were normalized in MG RAST with a log-based transformation [*Y*_s,i_ = log_2_ (*X*_s,i_ + 1)], where *X*_s,i_ represents an abundance measure (*i*) in sample (*s*). Log transformed counts from each sample were then standardized (data centering) according to the equation [*Z*_s,i_ = (*Y*_s,i_ − *Y*_s_)/ σ_s_)], where *Z*_s,i_ is the standardized abundance of an individual measure *Y*_s,i_ (log transformed from previous equation). From each log transformed measure of (*i*) in sample (*s*), the mean of all transformed values (*Y*_s_) is subtracted and the difference is divided by the standard deviation (σ_s_) of all log-transformed values for the given sample. After log transformation and standardization, the values for the functional categories within each sample were scaled from 0 (minimum value of all samples) to 1 (maximum value of all samples), which is a uniform scaling that does not affect the relative differences of values within or between samples. The top 100 processes which differed most substantially in the level of gene expression between frozen and thawed samples and which passed the One-Way ANOVA test were used as input for heatmap presentation, hypothetical proteins and proteins of unknown function were removed from the list of top 100 processes after creating the heatmap.

## Results and discussion

### Composition of permafrost SOM

SOM composition, as indicated by bulk metrics (TOC, TN, δ^13^C, δ^15^N) and lipid biomarkers (fatty acids [FAs] and alkanes), reflected mainly higher plant sources (Figure 1). %TOC and %TN were fairly constant throughout the soil core, with maximum values at 33.5 cm depth (Figure 1A). Elevated %TOC at 33.5 cm suggests that this horizon represents the permafrost table, assuming that cryoturbation under the tussock tundra and dwarf shrub vegetation has caused frost-churning of organic matter into the underlying mineral soil horizons, which then concentrated in the upper permafrost (Ping et al., 1997). Bulk soil δ^13^C signatures ranged from −26 to −27‰, reflecting inputs from C_3_ plants (Figure 1A). Soil δ^15^N was most enriched in the deepest horizon (Figure 1A), which could be indicative of several processes including preferential mineralization and plant uptake of isotopically light nitrogen compounds, microbial production of ^15^N enriched compounds during decomposition, and contributions from mycorrhizal fungi (Hogberg, 1997; Hobbie and Hobbie, 2008). The permafrost table was most depleted in δ^15^N, suggesting a lower degree of decomposition of plant organic matter than in the deeper soil horizons (Andersson et al., 2012). Concentrations of total alkanes and FAs were generally highest in the deepest soil horizon and lower at shallower depths. Long chain FAs [even Σ (C_24:0_-C_28:0_)] (Killops and Killops, 2005) and alkanes [Σ (C_22:0_-C_33:0_)] (Yunker et al., 1995) characteristic of vascular plants were important constituents of the total FA and alkane pools, accounting for 42–51 and 56–82%, respectively (Figure 1B). The CPI_alkane_ was >5, ranging from 5.17 at 49 cm to 7.75 at 126 cm, indicating that inputs from higher plants were important. The P_wax_ and P_aq_ indices suggested that contributions from higher plants were high while inputs from aquatic macrophytes were low at all depths except for 49 and 126 cm. In combination, the FAs and n-alkanes suggested that higher plants were the main source of SOM and likely deposited under drier conditions. However, aquatic macrophytes and mosses may have been important sources of SOM 2900 and 4100 calendar years before the present (cal. a BP) (Figure 1B) and conditions may have been wetter during those periods.

Lipid biomarkers reflecting bacterial inputs and processes indicated microbial decomposition occurred throughout the core. Contributions from bacterial FAs [iso-, anteiso- odd Σ (C_13:0_-C_17:0_)] (Meyers, 2003) were similar across depth horizons but were greatest at 126 cm (Figure 1B). Microbial decomposition of SOM occurred in all of the soil horizons, as indicated by the presence of Olean-13(18)-ene and Olean-12-ene, which are diagenetic products of higher plants (Yunker et al., 1995), and the ratio of branched C_15_:C_14_ FAs (Lu and Meyers, 2009) (Figure 1B). Since iso- and anteiso-C_15_ compounds are produced by bacteria from straight chain C_14_ precursors, an increase in the ratio of branched C_15_: straight chain C_14_ compounds may reflect organic matter decomposition (Lu and Meyers, 2009). Thus, SOM decomposition may be most extensive in the deepest soil horizon where branched C_15_:C_14_, contributions from bacterial FAs, and concentrations of total FAs and alkanes were the highest.

Overall, these results indicate that SOM was largely derived from vascular plants and microbial decomposition occurred throughout the soil column. Microbial breakdown of SOM may have occurred during deposition or after the soils were frozen, but we cannot distinguish between these possibilities based on the lipid biomarker results.

### Overview of the metatranscriptomic datasets

An average of 29,022 contigs (SD = 8951) with an average length of 616 (*SD* = 52) were assembled *de-novo* from each Illumina-sequenced metatranscriptome, with 87% (*SD* = 3%) of original reads mapping to contigs per sample (Supplementary Table S1). Open reading frames (ORFs) on contigs were assigned to COG (Cluster of Orthologous Genes) functional classes and high-level taxonomic groups (Class level and above). Transcribed ORFs with homology to proteins representing 22 out of the 25 defined Clusters of Orthologous Genes (Tatusov et al., 1997) were detected in metatranscriptomes from both frozen and thawed permafrost. Similar to a previous study utilizing the same cDNA amplification and sequence classification procedures (Orsi et al., 2013), the majority of reads could be annotated (86 ± 6%) in each sample, with the majority (79 ± 7%) of annotated reads coding for proteins and a minimal representation (7 ± 2%) of ribosomal RNA reads (Supplementary Table S1).

### Overexpressed genes under frozen conditions

**Figure 2:**
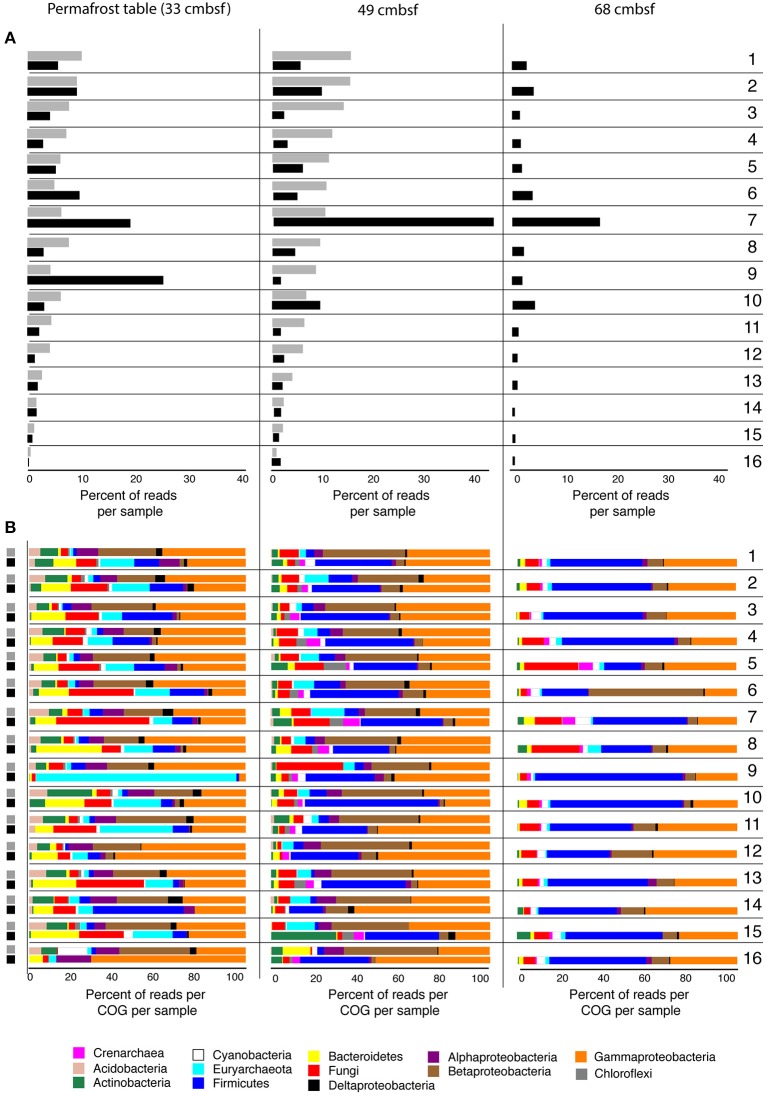
**(A)** Relative abundance of reads assigned to COGs in metatranscriptomes from frozen (gray bars) permafrost soils of core TL08S1C3 at 33.5 ± 2 cm (permafrost table) and 49 ± 2 cm depth and at 33.5 ± 2, 49 ± 2, and 68 ± 2 cm after 11 days of incubation at 4°C (black bars). Only the 16 most (relative) abundant COGs are shown (excluding [R] General function prediction and [S] Function unknown). COGs detected in low abundance, but not shown, are [A] RNA processing and modification; [V] Defense mechanisms; [Z] Cytoskeleton. **(B)** The prokaryotic and fungal ORFs as percent of reads per COG per sample with homology to the following COG families: **1**, Amino acid transport and metabolism; **2**, Energy production and conversion; **3**, Inorganic ion transport and metabolism; **4**, Replication, recombination and repair; **5**, Transcription; **6**, Posttranslational modification, protein turnover, chaperones; **7**, Translation, ribosomal structure and biogenesis; **8**, Cell wall/membrane/envelope biogenesis; **9**, Coenzyme transport and metabolism; **10**, Carbohydrate transport and metabolism; **11**, Signal transduction mechanisms; **12**, Lipid transport and metabolism; **13**, Nucleotide transport and metabolism; **14**, Intracellular trafficking, secretion, and vesicular transport; **15**, Cell cycle control, cell division, chromosome partitioning; **16**, Cell motility.

Amino acid transport and metabolism, energy production, and DNA repair, replication and recombination were amongst the most relatively abundant COG categories expressed in the frozen permafrost (Figure 2A). The expression of genes corresponding to most major COG families in frozen permafrost suggests the *in situ* activity of microbes, even at subfreezing temperatures. The variability in relative abundances of COGs was markedly less in metatranscriptomes from frozen samples, compared to the metatranscriptomes from thawed permafrost (Figure 2A) Phylogenetic binning indicates that Proteobacteria (γ, β, α, and δ), Firmicutes, Acidobacteria, and Actinobacteria, as well as Euryarchaeota and ascomycetous Fungi were the most transcriptionally active microbial groups under frozen conditions in the permafrost table (Figure 2B). These taxa are frequently reported from Arctic soils (Jansson and Tas, 2014 and references therein).

**Figure 3:**
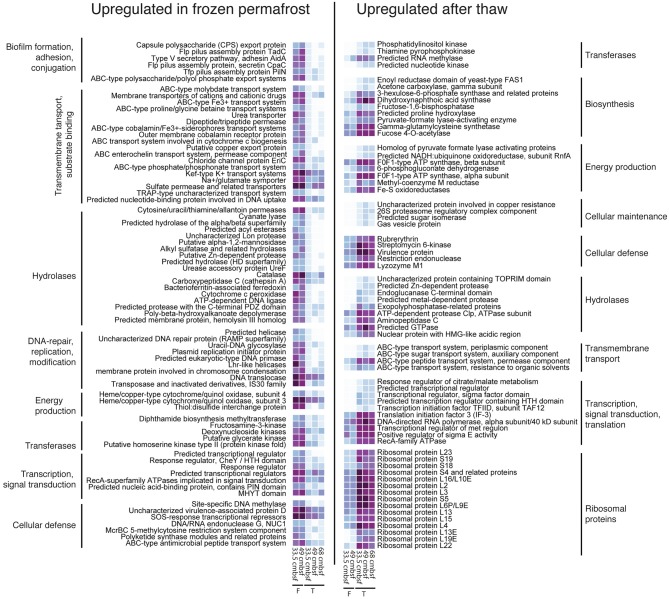
**Heatmap showing the most abundant overexpressed genes in frozen and thawed permafrost soils**. Shown is the distribution of normalized reads mapping to contigs with homologous ORFs (>60% amino acid identity) to proteins in the COG database. The distribution of functional categories and the colors correspond to log transformed read abundance (dark purple is more, light blue is less).

Several transcripts encoding proteins involved in biofilm formation, virulence, and horizontal gene transfer had relatively higher expression levels under frozen conditions (Figure 3). Notably, expression of genes coding for pilus assembly proteins (COGs 2064, 1706, 4964, 3166) may indicate biofilm formation, which is a common feature among bacteria living under stressful conditions and causes a close interaction between cells that can promote virulence, bacterial signaling, and/or horizontal gene transfer (Varga et al., 2008). Biofilm formation in the frozen soils may be related to the small area available for growth in the permafrost microhabitat where the current consensus is that microbes inhabit very thin liquid brine veins surrounding frozen soil particles (Gilichinsky et al., 2003, 2005; Shcherbakova et al., 2005; Onstott et al., 2009; Pecheritsyna et al., 2012). Active defense against viral infection is indicated by the activity of a Type II restriction-modification system (i.e., site-specific DNA methylase COG0338; Figure 3), which can protect bacteria and archaea against invading foreign DNA (Pingoud et al., 2005). However, virulence-associated genes were also overexpressed in thawed soils, suggesting active viral defenses during both frozen and thawed conditions. Enzymes involved in DNA repair were also overexpressed in frozen soils (Figure 3). Uracil-DNA glycolases are involved in base excision repair, a process that removes damaged bases that could otherwise lead to mutations (Kim and Wilson, 2012). Recently, an ionization radiation (IR) experiment with a frozen culture of *Psychrobacter arcticus* suggested that, in the presence of long-term natural background IR, permafrost bacteria can repair double stranded DNA breaks in the absence of net growth (Dieser et al., 2013). Our results support this observation, and suggest that *in situ*, uracil-DNA glycolases play an important role in DNA repair incurred from long-term exposure to natural background IR sources in the permafrost environment. Compared to thawed soils, a gene sequence encoding a *Rec*A-mediated autopeptidase was relatively more abundant in the permafrost sequence datasets further suggesting that bacterial “Save Our Ship” (SOS)-response occurs in the frozen soils (Figure 3). SOS-response is a global response to DNA damage in which the cell cycle is arrested and DNA repair is induced (Michel, 2005). Such DNA repair and stress response mechanisms likely allow microbes to survive over geological timescales in permafrost until favorable conditions for growth are experienced, such as thawed conditions resulting from increasing atmospheric temperatures.

### Overall transcriptional activity stimulated under thawed conditions

Compared to the frozen samples, all three thawed permafrost intervals exhibit relatively increased representation of transcripts involved in translation, ribosomal structure, and biogenesis, indicating a general increase in microbial activity and growth (Figure 2A). In thawed samples from the permafrost table, metatranscriptomes had an increased proportion of transcripts involved in coenzyme transport and metabolism compared to metatranscriptomes from frozen permafrost table samples (Figure 2A). The majority of transcripts falling into this functional category in permafrost table metatranscriptomes were assigned to Euryarchaeota (Figure 2B).

Overall, there was increased representation of Firmicutes, Bacteroidetes, Euryarchaeota, and ascomycetous Fungi in metatranscriptomes from thawed samples relative to Proteobacteria, Acidobacteria, and Actinobacteria (Figure 2B). The thawed permafrost table at 33.5 cm showed the largest relative increase in fungal-derived transcripts, compared to the frozen sample from this horizon. The thawed horizon at 49 cm, revealed the largest relative increase in transcripts assigned to Firmicutes compared to frozen permafrost at this depth, and a similar proportion of transcripts were assigned Firmicutes in thawed samples from 68 cm (Figure 2B). This may be due to the germination of spores in permafrost soils after thawed conditions (Steven et al., 2008; Niederberger et al., 2009). Transcripts assigned to Chloroflexi and Crenarchaeota involved in translation, ribosomal structure and biogenesis at 49 cm increased in relative abundance in metatranscriptomes from thawed samples (Figure 2B). Similar proportions of transcripts were assigned to Chloroflexi and Crenarchaeota in metatranscriptomes from thawed permafrost at 68 cm. Transcriptional activity of these phyla mainly increased in the deeper thawed soils, suggesting that they may be specialized in the initial decomposition of more complex, ancient SOM.

Relative to the frozen samples, thawed metatranscriptomes exhibit an upregulation of genes coding for enzymes involved in extracellular protein degradation (notably Aminopeptidase C) (Figure 3). This suggests microbial utilization of labile proteins as a source of N and C during thawed conditions. Thawed metatranscriptomes also had a relative increase in transcripts encoding proteins involved in uptake, transport, and degradation of carbohydrates (e.g., ABC type sugar transporter, a predicted sugar isomerase, 6-phosphogluconate dehydrogenase, fructose 1,7 biphosphatase, and a pyruvate formate lyase-activating enzyme) (Figure 3). Upon thawed conditions, some of the microbial metabolism appears to be anaerobic, as evidenced by the relative increased expression of the pyruvate-formate lyase-activating enzyme (Figure 3), which catalyzes the anaerobic conversion of pyruvate and coenzyme A into acetyl CoA and formate (Jiang et al., 2001).

### Transcriptional evidence for enzymatic decomposition of complex soil biopolymers under thawed conditions

**Figure 4:**
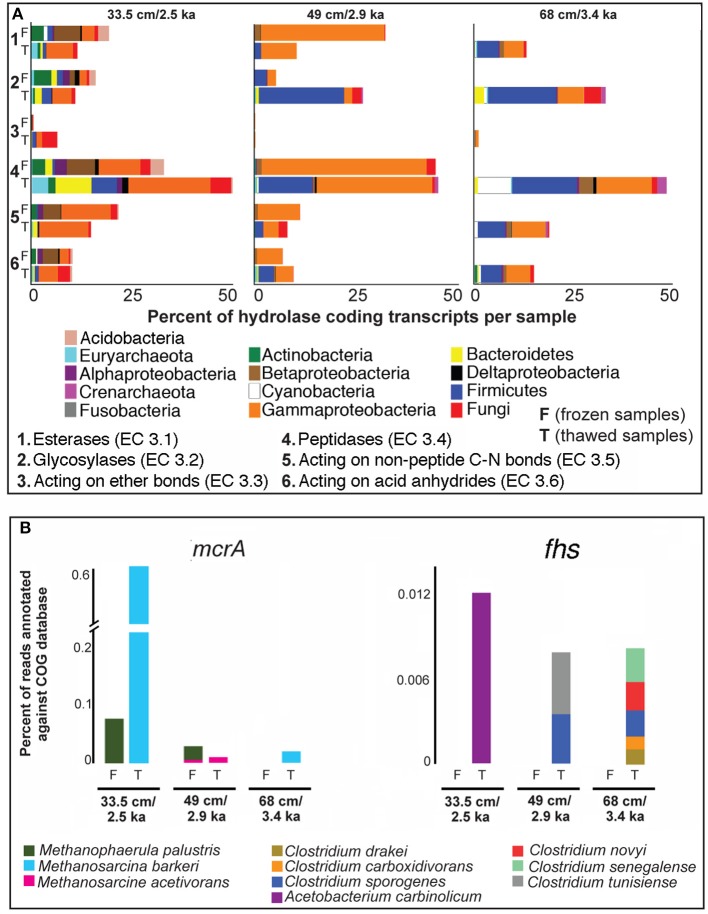
**Expressed homologs, (relative abundance) encoding (A) hydrolases potentially involved in the initial breakdown of of complex permafrost soil biopolymers, and (B) formyltetrahydrofolate synthetase (*fhs*) and methyl-coenzyme reductase A (*mcrA*) before and after 11 days of thaw**. Note the predominance of concomitant *mcr*A (methanogenesis) and *fhs* (acetogenesis) expression in thawed samples relative to frozen samples.

Genes encoding for all major classes of hydrolases involved in the cleavage of complex biopolymers into C1 and C2 substrates were expressed in both frozen and thawed soils (Figure 4A). The relative distribution of hydrolase-encoding transcripts at the shallowest interval (permafrost table), revealed a relative increase in peptidase encoding transcripts in thawed samples (Figure 4A). This difference was not observed for the deeper permafrost sample (49 cm), and may indicate increased bioavailability of peptides at 33.5 cm after thaw. Most of this relative increase in peptidase encoding transcripts at 33.5 cm could be attributed to the activity of euryarchaea and bacteroidetes (Figure 4A). This confirms our earlier findings based on detectable activities of leucine aminopeptidase at this depth interval, indicating that the permafrost table contains relatively labile polypeptides or proteins as source of C and N (Coolen et al., 2011).

The diversity of taxa expressing hydrolase encoding transcripts in the deeper permafrost decreased markedly relative to 33.5 cm, with Firmicutes-derived hydrolase encoding transcripts increasing in relative representation after thaw (Figure 4A). A similar response was observed for crenarchaeal transcripts encoding glycosylases and peptidases, which were only observed in the two deepest thawed permafrost intervals (Figure 4A). Despite the overall increase in activity of Chloroflexi in the thawed soil interval at 49 cm (Figure 2B), transcripts homologous to Chloroflexi–derived hydrolases acting on complex soil biopolymers were not observed early after thaw (Figure 4A). Their role in the cycling of organic matter in permafrost soils requires further investigation.

Upon thawed conditions in the permafrost table, relative expression of fungal, γ-Proteobacterial, and Firmicutes genes encoding hydrolases acting on recalcitrant ether bonds increased (Figure 4A). Ether bonds occur in the tetraether membranes of yet unidentified soil bacteria (i.e., branched Glycerol Dialkyls Glycerol Tetraethers [branched GDGTs]), commonly found in peat soils (Weijers et al., 2007), as well as in isoprenoid GDGTs of (methanogenic) archaea (Schouten et al., 2000). The increased transcription of genes encoding hydrolases acting on ether bonds in the permafrost table upon thaw suggests that some permafrost microbiota at 33.5 cm decompose dead bacterial and/or archaeal biomass. Collectively these data suggest that thawed conditions stimulate growth and turnover of archaeal and bacterial biomass in soil permafrost.

Relative expression of genes with homology to cyanobacterial peptidases and hydrolases acting on non-peptide C-N bonds and anhydrides was highest in the deepest analyzed thawed permafrost interval (Figure 4A). The activity in permafrost soils in the absence of light seems counter-intuitive. However, oligopeptides and or amino acids may serve as nitrogen sources for cyanobacteria in permafrost soils, as they have been reported to assimilate organic compounds for biosynthesis during the dark phase of their life cycle (Vernotte et al., 1992). Lay et al. (2013) reported the presence of cyanobacterial genes involved in the reduction of nitrate to ammonia in high-Arctic saline subzero spring sediments, and they suggested that this strategy might be advantageous for maintaining activity during the long-term darkness of the Arctic winter. Our results suggest that cyanobacteria may be dormant in the deeper permafrost soils for substantial periods of time and can regain activity under dark conditions within days after thaw.

### Active metabolic pathways for methanogenesis and acetogenesis stimulated under thawed conditions

**Figure 5:**
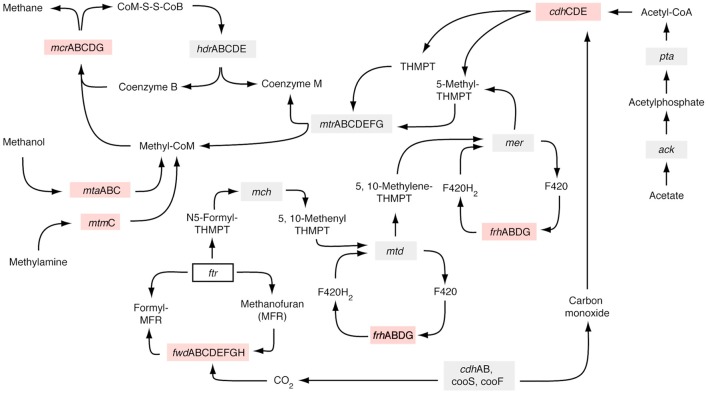
**Metabolic pathways actively expressed by the methanogen *Methanosarcina barkeri* in metatranscriptomes from frozen and thawed permafrost samples**. The *M. barkeri* genome was chosen as a reference because it was found to be the dominant methanogen expressing *mcr*A after thaw (Figure 4B). Red boxes represent expressed genes that were only detected after thaw, gray boxes represent genes expressed before and after thaw, and clear boxes are genes that were not detected under any conditions. **CoM-S-S-CoB**, Coenzyme M 7-mercaptoheptanoylthreonine-phosphate heterodisulfide; **THMPT**, Tetrahydromethanopterin; **hcr**, Heterodisulfide reductase subunit A; **mcr**, Methyl-coenzyme reductase; **mtr**, Tetrahydromethanopterin S-methyltransferase; **CoM**, Coenzyme M; **mtm**, Monomethylamine corrinoid protein; **mta** Methanol-specific corrinoid protein; **cdh**; Acetyl-CoA decarbonylase/synthase complex; **frh**, Coenzyme F420 hydrogenase; **mer**, 5,10-methylenetetrahydromethanopterin reductase; **mch**, Methenyltetrahydromethanopterin cyclohydrolase; **mtd**, Methylenetetrahydromethanopterin dehydrogenase; **fwd**, Formylmethanofuran dehydrogenase; **pta**, Phosphate acetyltransferase; **ack**, Acetate kinase; **cooS/F**, Carbon monoxide dehydrogenase.

Metatranscriptomes from thawed permafrost exhibited a marked relative increase in transcripts involved in coenzyme transport and metabolism (Figure 2A). In the permafrost table, metatranscriptomes in this category were mainly assigned to Euryarchaeota (Figure 2B). The most predominant transcript in this category was methyl coenzyme M reductase subunit A (*mcr*A), which catalyzes the last step in methanogenesis and is present in all methanogens (Ferry, 1999). More specifically, the *mcr*A transcripts in the thawed permafrost table were assigned to the versatile methanogen *Methanosarcina barkeri*, capable of using a variety of C_1_ and C_2_ compounds including acetate (Weimer and Zeikus, 1979; Jetten et al., 1992). The identification of *M. barkeri* as the main producer of *mcr*A transcripts is plausible since acetate concentrations in permafrost samples can be 50 times higher (Strauss et al., 2012) than the minimum acetate requirement (~200 μM) of *Methanosarcina* spp. (Jetten et al., 1992). Expression of methano phenazine-reducing hydrogenase (*mph*), which is indicative of heterotrophic methanogenic metabolism, was also detected after thaw, suggesting C1 and C2 substrates as sources of biogenic methane. Further metabolic mapping of expressed genes to the *M. barkeri* genome indicated an increase in active biochemical pathways utilizing acetate, trimethylamine, and methanol as methanogenic substrates (Figure 5). Expression of genes coding for formylmethanofuran-tetrahydromethanopterin N-formyltransferase (*ftr*) (a protein necessary completing the pathway from methanogenesis from CO_2_), was not detected, suggesting a predominance of heterotrophic methanogenesis after thaw. A marked increase in acetoclastic methanogensis was recently also reported from thawed permafrost soils in northern Sweden (McCalley et al., 2014). In addition, recent biogeochemical examination of peat and dissolved organic matter (DOM) at northern Sweden permafrost sites that had been exposed to thaw for up to 40 years revealed a shift in CH_4_ production pathway from CO_2_-reduction to acetate cleavage, which was associated with (i) increased humification rates, (ii) DOM shifted toward lower molecular weight compounds with lower aromaticity, (iii) lower organic oxygen content, and (iv) more abundant microbially produced compounds (Hodgkins et al., 2014).

In all thawed permafrost samples, an increase in *mcr*A expression was accompanied by an increase in expression of formyltetrahydrofolate synthetase (*fhs*) genes (not detected in frozen permafrost), which can serve as a proxy for acetogenesis (Lever, 2013) (Figure 4B). Transcripts encoding *fhs* in the upper permafrost were assigned predominantly to *Acetobacterium carbinolicum*, whereas various *Clostridium* species are likely involved in acetate production in deeper permafrost (Figure 4B). The genetic expression of the acetate-utilizing methanogenesis pathway (Figure 5) after thaw suggests that some of this acetate is then utilized as a substrate by methanogens, predominantly *M. barkeri* (Figure 4B). Acetogenic fermentation, which is also likely under anaerobic conditions in thawed permafrost, could be another source of acetate for acetoclastic methanogens. Bacterial genes encoding the two enzymes diagnostic for fermentative conversion of acetyl-CoA to acetate (acetate kinase, EC:2.7.2.1 and phosphotransacetylase EC:2.3.1.8) were not detected in thawed permafrost, suggesting that pyruvate fermentation to acetate may not be a dominant source of acetate, relative to homoacetogenesis. However, to substantiate the claim of homoacetogenesis dominating over fermentation as a source of acetate in thawing permafrost soils, it is necessary to perform radiotracer incubations or to analyze the stable isotopic composition of acetate in these soils (Rivkina et al., 2000; Bakermans and Skidmore, 2011).

## Concluding remarks

Our pilot study shows that the analysis of metatranscriptomes is a sensitive approach capable of identifying a wide spectrum of important metabolic processes in frozen and thawed permafrost. In general, the transcriptional profile under frozen conditions suggests a dominance of stress responses, survival strategies, and maintenance processes whereas upon thaw a rapid enzymatic response to decomposing SOM was observed. Furthermore, our analysis indicates that acetoclastic methanogenesis may be a dominant form of methanogenesis in thawing permafrost soils fueled, in part, by acetate production from acetogenic bacteria. Due to large heterogeneity in permafrost SOM and microbial composition, future metatranscriptome studies should be performed from a wider variety of permafrost soil types and locations, and in comparison with the active layer. Future studies comparing metatranscriptomes from thawing permafrost should also contain biological replicates to provide robust statistical power for hypothesis testing. Furthermore, paired geochemical analysis of isotopically labeled substrates and metabolites during thaw incubation experiments as well as the analysis of the stable isotopic composition and concentration of dissolved methane should be performed to validate mRNA-based inferences of metabolic pathways. Measurements of soil pH would further be useful to contextualize microbial responses to permafrost thaw at the transcriptional level. Metatranscriptomics is shown here to be a useful approach for inferring the activity of permafrost microbes, that has potential to improve our understanding of permafrost SOM bioavailability and biogeochemical mechanisms contributing to greenhouse gas emissions as a result of warming induced permafrost thaw.

### Conflict of interest statement

The authors declare that the research was conducted in the absence of any commercial or financial relationships that could be construed as a potential conflict of interest.
