# Supplementary material for: The transcriptional response of microbial communities in thawing Alaskan permafrost soils
Source: Front Microbiol. 2015 Mar 16;6:197. doi: 10.3389/fmicb.2015.00197 (PMC4360760; doi:10.3389/fmicb.2015.00197)
Supplement: Supplementary file 1 [file Table1.DOC]

**Supplementary Table S1.** Annotation statistics of the analyzed frozen and thawed permafrost transcriptomes.
